# Effects of free-position delivery combined with perineal massage on reducing sensitive indicators of care quality in high-risk pregnant women

**DOI:** 10.3389/fmed.2025.1661126

**Published:** 2025-12-17

**Authors:** Ying Shen

**Affiliations:** The Affiliated Hospital of Jiangnan University, Wuxi, Jiangsu, China

**Keywords:** free-position delivery, perineal massage, high-risk pregnancy, vaginal delivery, perineal injury, maternal outcomes

## Abstract

**Aims:**

The aim of this study was to evaluate the impact of free-position delivery combined with perineal massage on nursing-sensitive quality indicators and clinical outcomes in high-risk pregnant women.

**Methods:**

This retrospective cohort study analyzed clinical data from 223 high-risk pregnant women who underwent vaginal delivery between January 2023 and December 2024. Participants were allocated to either the Traditional Supine Delivery group (TSD, *n* = 117) or the Free-Position and Perineal Massage group (FPPM, *n* = 106) based on delivery mode. Baseline characteristics, durations of labor, perineal injuries, postpartum hemorrhage, anesthesia requirements, postpartum complications, neonatal conditions, psychological status, and maternal satisfaction were compared.

**Results:**

Compared with TSD, the FPPM group had shorter active first stage and second stage of labor (5.98 ± 1.25 vs. 6.33 ± 1.21 h; 82.01 ± 8.14 vs. 85.48 ± 7.85 min; both *p* < 0.05), and a lower rate of perineal lacerations (59.43% vs. 76.07%; *p* = 0.008), episiotomy (16.04% vs. 27.35%; *p* = 0.042), and postpartum hemorrhage ≥500 mL (5.66% vs. 13.68%; *p* = 0.045). FPPM also reduced intrapartum anesthesia requirements and postpartum complications while improved 5-min Apgar scores (9.80 ± 0.35 vs. 9.69 ± 0.42; *p* = 0.036), maternal satisfaction (69.81% vs. 52.14% very satisfied; *p* = 0.026), and PTSD symptoms (28.44 ± 2.31 vs. 29.52 ± 2.86; *p* = 0.002).

**Conclusion:**

Free-position delivery combined with perineal massage in high-risk pregnant women is associated with better nursing-sensitive quality indicators, lower rates of perineal trauma and postpartum complications, improved neonatal outcomes, and higher maternal satisfaction compared with traditional supine delivery.

## 1 Introduction

High-risk pregnancies and deliveries continue to pose major clinical challenges, significantly contributing to maternal and neonatal morbidity and mortality rates worldwide (1). High-risk pregnant women (i.e., those with advanced maternal age, hypertensive disorders, diabetes, cardiac disease, malnutrition, or other comorbidities) present unique challenges during labor and birth, necessitating vigilant maternal and fetal monitoring, tailored interventions, and heightened standards of care (2). The primary goal for these women is not only to improve survival rates but also to enhance the overall birth experience and health outcomes by optimizing sensitive quality indicators of obstetric care.

Obstetric care quality sensitive indicators refer to measurable outcomes that directly reflect the effectiveness, safety, and patient-centeredness of nursing interventions. These include nursing satisfaction, episiotomy rates, postpartum hemorrhage status, wound infection rates, etc., all of which are significantly influenced by nursing practices (2). Traditional labor management practices, particularly the routine use of the supine or lithotomy position and lack of proactive perineal protection, may inadvertently contribute to increased intervention rates and poorer birth experiences, especially among high-risk pregnant women (3). Supine positioning may compromise uterine perfusion, limit the physiological advantage of gravity, inhibit the mother’s ability to move spontaneously, and increase the risk of prolonged labor, operative intervention, and perineal injury (4). Recent practice guidelines and research studies emphasize the value of person-centered and physiologically supportive care models, highlighting evidence for alternative birthing positions, maternal autonomy, and non-pharmacological approaches for reducing morbidity and improving satisfaction (3, 4).

Free-position delivery, a care model allowing pregnant women to adopt positions of their choice during labor and delivery, has garnered attention as a promising strategy to enhance labor progress, decrease pain, and facilitate spontaneous vaginal delivery (5). At the same time, perineal massage during the late first or second stage of labor, aimed at improving perineal elasticity and circulation, has been shown to reduce the frequency and severity of perineal lacerations and the need for episiotomy (6). Although both interventions have individually demonstrated benefits, there is a lack of robust data on their combined application in high-risk populations and their collective impact on critical care quality indicators, such as rates of perineal trauma, hemorrhage, anesthesia requirements, and psychosocial outcomes (7).

Sensitive indicators of care quality in obstetrics have evolved to encompass a broad range of maternal and neonatal outcomes, including not only clinical endpoints but also patient-reported experiences, satisfaction, pain management, and psychological well-being (8). In high-risk groups, where adverse outcomes are more prevalent, these indicators serve as vital benchmarks for evaluating and refining clinical practice (9).

Given the increasing emphasis on patient-centered care and the necessity for evidence-based, minimally invasive interventions in high-risk obstetric populations, further evaluation of combined non-pharmacological interventions is warranted. The present study was designed to investigate the effects of free-position delivery combined with perineal massage on sensitive indicators of care quality in high-risk pregnant women. By exploring both clinical and experiential outcomes, this study aims to provide data to inform best practices in the care of women with high pregnancy risk, thus contributing to the evolving paradigm of high-quality, patient-centered maternity care.

## 2 Materials and methods

### 2.1 Research design and ethical statement

This study is a retrospective cohort study. It analyzed the data of 223 pregnant women who underwent natural delivery at the Affiliated Hospital of Jiangnan University (Jiangsu, China) from January 2023 to December 2024. All 223 pregnant women were high-risk. Patient data were selected after meeting the inclusion and exclusion criteria. The grouping was entirely based on the actual protocols received during their hospital stay for delivery. Based on the mode of delivery, participants were divided into two groups: Traditional Supine Delivery group (TSD group) and Free-Position and Perineal Massage group (FPPM group). The TSD group included 117 patients, while the FPPM group included 106 patients.

This study was a retrospective analysis of completed patient cases, which does not affect the treatment or subsequent care of the patients. Furthermore, the data used was anonymized, ensuring that individual patient identities could not be traced back from the case data. For this reason, the study received approval from the Ethics Committee of the Affiliated Hospital of Jiangnan University and was exempted from the requirement for informed consent. The ethical approval number is LS2025283.

The primary outcome measures of this study aim to evaluate the core clinical effects of the intervention, including perineal injury and the incidence of postpartum hemorrhage. Secondary outcome measures are used to comprehensively assess the additional benefits and safety of the intervention, covering the duration of labor, intrapartum anesthesia requirements, postpartum complications, neonatal status, and postpartum psychological state and maternal satisfaction with care.

### 2.2 Grouping criteria

In this study, the TSD group used the traditional supine position for delivery and did not receive perineal massage. On the other hand, the FPPM group used a free-position delivery approach and received perineal massage during the second stage of labor, administered by trained midwives. The assignment of participants to groups was primarily based on their autonomous choice before delivery. Each mother was informed of the advantages and disadvantages of both delivery methods before making a decision based on her personal preference.

To ensure the standardized implementation of the intervention, all midwives received uniform specialized training. The training content included a 4-h theoretical course and a 2-h practical drill, focusing on the physiological basis of free-position delivery, guidance techniques for different delivery positions, and the standard operating procedures for perineal massage. After the training, simulated assessments were conducted to ensure that each midwife mastered the proper operational skills. In practice, midwives strictly adhered to the hospital’s “Standard Operating Procedures for Free-Position Delivery and Perineal Massage,” which detailed the specific methods for position guidance, the steps for performing perineal massage, and relevant precautions.

The method of perineal massage involves performing the procedure when the cervix is nearly fully dilated (8–10 centimeters) or at the beginning of the second stage of labor. The midwife, wearing sterile gloves and using a water-soluble lubricant, inserts the index finger and thumb into the vagina and applies uniform, sustained pressure toward the rectum along the posterior vaginal wall. A U-shaped massage motion is performed, focusing on stretching the perineal body. Each massage session lasts approximately 5–10 min and can be repeated 2–3 times during the intervals between contractions, depending on the progress of labor and the mother’s tolerance, until the fetal head crowns. During the procedure, the midwife closely monitors the mother’s response and adjusts the pressure as needed to ensure comfort.

### 2.3 Inclusion and exclusion criteria

In this study, the inclusion criteria were as follows: (1) high-risk pregnant women meeting any one of the following conditions: age over 35 years, hypertension, diabetes, heart disease, malnutrition, thyroid disorders (10); (2) singleton pregnancy; (3) cephalic presentation; (4) planned vaginal delivery; (5) cervical ripeness (Bishop score ≥ 6); and (6) complete data available.

The exclusion criteria included: (1) pregnant women with multiple high-risk factors; (2) multiple pregnancies; (3) breech or transverse presentation; (4) planned cesarean section or conversion to cesarean section during labor; (5) extremely poor perineal conditions (previous third- or fourth-degree lacerations, severe vaginitis, or infections); (6) severe internal medical conditions or other significant comorbidities; (7) pregnant women who smoke, consume excessive alcohol, or have a history of substance abuse; (8) history of uterine surgery such as myomectomy; (9) infectious diseases such as HIV/AIDS, hepatitis B, hepatitis C, syphilis, etc.; (10) patients with severe mental or cognitive impairments unable to comply with follow-up and assessment; and (11) missing data.

### 2.4 Baseline data

Baseline data and demographic information were recorded in the medical record system during the initial consultation. Fetal weight was measured immediately after birth. The amniotic fluid index (AFI) was assessed using an ultrasound device (Voluson E10, GE Healthcare, United States) by measuring the maximum vertical depth of the largest fluid pocket in each of the four quadrants of the uterus (upper left, upper right, lower left, lower right). The sum of these four measurements constitutes the AFI. Prior to delivery, a vaginal examination was performed to evaluate cervical ripeness using the Bishop score (11).

### 2.5 Duration of labor

The duration of labor was fully documented by nursing staff and divided into the active phase of the first stage, the second stage, and the third stage of labor. The time for each stage was retrieved from the medical record system for analysis.

### 2.6 Perineal injury

After delivery, the degree of perineal injury was assessed by physicians, with injuries classified as follows: Grade I injuries are limited to the perineal skin or vaginal mucosa; Grade II injuries involve the perineal skin, vaginal mucosa, and some muscles of the perineal body (such as the bulbospongiosus muscle and superficial transverse perineal muscle) but do not involve the external anal sphincter; Grade III injuries extend to the perineal skin, vaginal mucosa, muscles of the perineal body, and the external anal sphincter; Grade IV injuries involve the perineal skin, vaginal mucosa, muscles of the perineal body, external anal sphincter, and rectal mucosa, completely penetrating the rectal wall (12). The occurrence of mediolateral episiotomy during delivery was documented by healthcare staff in the surgical records.

### 2.7 Postpartum hemorrhage within 24 h

Postpartum hemorrhage (PPH) was defined as blood loss exceeding 500 milliliters within the first 24 h after delivery. The amount of blood loss was calculated by weighing the sanitary pads, gauze, and perineal pads used by the parturient during the first 24 h postpartum. On the second day after delivery, fasting venous blood samples were collected into EDTA anticoagulant tubes (Vacutainer, Becton Dickinson, USA) and analyzed for hemoglobin levels using a fully automated hematology analyzer (Sysmex XN-1000, Sysmex Corporation, Japan). Intervention Needed was defined as the use of an intrauterine balloon, embolization, surgery, or transfer to the intensive care unit (ICU).

### 2.8 Anesthesia requirements

Intrapartum pain levels were assessed using the Visual Analog Scale (VAS) (13). The decision to administer additional anesthesia was made by the physician based on the patient’s pain status and the progress of labor. Local anesthesia may be required if a mediolateral episiotomy is performed. Instances where additional anesthesia was administered during the procedure were documented in the surgical records.

### 2.9 Postpartum complications

Postpartum, nursing staff monitored patients for symptoms of puerperal infection, urinary tract injury, and urinary retention throughout the entire hospital stay. These monitoring measures help in the early detection and management of potential postpartum complications, ensuring patient safety and recovery.

### 2.10 Neonatal condition

The Apgar score was used to assess the neonatal condition (14). Blood samples were collected from the umbilical vein into heparinized tubes (Vacutainer, Becton Dickinson, United States) and analyzed for pH using a portable blood gas analyzer (i-STAT, Abbott, United States). Statistical analysis was performed on the incidence of mild to moderate asphyxia, and no severe asphyxia cases were observed.

### 2.11 Patient psychological status

At 4 weeks postpartum, the Edinburgh Postnatal Depression Scale (EPDS) was used to screen for postpartum depression (15). The scale consists of 10 items, with each question having four response options, scored from 0 to 3. The total score ranges from 0 to 30, with higher scores indicating more severe depressive symptoms. A total score exceeding 10 suggests the possible presence of postpartum depression.

The Posttraumatic Stress Disorder Checklist for DSM-5 (PCL-5) is a widely used self-report measure for assessing the severity of posttraumatic stress disorder (PTSD) symptoms experienced by an individual over the past month (16). This scale includes 20 items, each rated on a 5-point scale ranging from 0 (not at all) to 4 (extremely), with a score greater than 33 indicating potential PTSD symptoms.

The scale was completed by the mothers in a quiet environment, with researchers providing unified instructions before distributing the scales.

### 2.12 Satisfaction

The Maternal Satisfaction with Obstetric Care Scale (MSOCS) was used to assess maternal satisfaction with delivery care services, covering various aspects of the birthing process, including healthcare provider attitudes, medical facility conditions, and information provision (17). The scale consists of 20 items; each rated on a scale from 1 (very dissatisfied) to 5 (very satisfied). A total score greater than 80 is defined as very satisfied, a score between 60 and 80 is defined as generally satisfied, and a score below 60 is defined as dissatisfied. This study utilized the Chinese version of the scale, which was ensured to be conceptually equivalent through a standard translation-back-translation process. In a pilot test, the Cronbach’s *α* coefficient was measured at 0.89, indicating good reliability.

### 2.13 Statistical analysis

In the data processing phase of this study, no missing data was identified. Data analysis was conducted using SPSS 29.0 statistical software (provided by SPSS Inc., Chicago, IL, United States). Categorical variables were presented as frequencies and percentages [*n* (%)] and evaluated using chi-square tests and relevant basic formulas. For continuous variables, the Shapiro–Wilk test was first applied to verify whether they followed a normal distribution. If continuous variables exhibited a normal distribution, they were reported as mean ± standard deviation (Mean ± SD) and group comparisons were performed using t-tests adjusted for analysis of variance. The result was considered statistically significant if the two-tailed *p*-value was less than 0.05.

Participants were re-grouped based on whether perineal lacerations occurred during delivery. A multivariate logistic regression analysis was then conducted to determine the association between the FPPM intervention and the incidence of perineal lacerations.

## 3 Result

### 3.1 Baseline data

Baseline demographic and clinical characteristics were comparable between the TSD group and the FPPM group (Table 1). These results indicate that both groups were well matched in terms of baseline characteristics, providing a valid foundation for subsequent comparative analyses.

**Table 1:** Baseline data.

Criterion	TSD group (*n* = 117)	FPPM group (*n* = 106)	*t*/*χ*^2^	*p*
Age	29.91 ± 2.29	29.69 ± 2.24	0.716	0.475
Pre-pregnancy weight	67.87 ± 7.84	68.19 ± 8.26	0.294	0.769
Height	1.63 ± 0.12	1.65 ± 0.11	1.216	0.225
Pre-pregnancy BMI			1.255	0.740
Underweight	17 (14.53%)	11 (10.38%)		
Normal	82 (70.09%)	81 (76.42%)		
vOverweight	13 (11.11%)	10 (9.43%)		
Obese	5 (4.27%)	4 (3.77%)		
High-risk factors			1.074	0.956
Age over 35	48 (41.03%)	41 (38.68%)		
Hypertension	39 (33.33%)	32 (30.19%)		
Diabetes	12 (10.26%)	13 (12.26%)		
Heart disease	5 (4.27%)	7 (6.60%)		
Malnutrition	10 (8.55%)	10 (9.43%)		
Thyroid disorders	3 (2.56%)	3 (2.83%)		
Residential area			0.101	0.751
Urban	85 (72.65%)	79 (74.53%)		
Rural	32 (27.35%)	27 (25.47%)		
Previous fetal loss	4 (3.42%)	3 (2.83%)	0	1.000
Baseline systolic blood pressure	122.67 ± 12.44	122.14 ± 11.88	0.325	0.746
Gestational age	37.16 ± 1.52	37.02 ± 1.24	0.767	0.444
Hemoglobin	126.74 ± 4.87	127.25 ± 4.49	0.814	0.417
Fetal weight	2896.94 ± 478.21	2976.25 ± 447.76	1.275	0.204
Amniotic fluid index	12.81 ± 1.22	12.84 ± 1.16	0.162	0.872
Bishop score	7.92 ± 0.80	7.88 ± 0.75	0.302	0.763

BMI, body mass index.

### 3.2 Duration of labor

The duration of labor differed significantly between the two groups. Compared with the TSD group (Table 2), the FPPM group had a shorter active first stage of labor (5.98 ± 1.25 vs. 6.33 ± 1.21 h; *p* = 0.033), a reduced second stage duration (82.01 ± 8.14 vs. 85.48 ± 7.85 min; *p* = 0.001), and a decreased total duration of labor (7.60 ± 1.42 vs. 8.01 ± 1.47 h; *p* = 0.033). No significant difference was observed in the duration of the third stage of labor between the groups (*p* = 0.945).

**Table 2:** Duration of labor.

Criterion	TSD group (*n* = 117)	FPPM group (*n* = 106)	*t*/*χ*^2^	*p*
Active first stage duration (hour)	6.33 ± 1.21	5.98 ± 1.25	2.142	0.033
Second stage duration (min)	85.48 ± 7.85	82.01 ± 8.14	3.241	0.001
Third stage duration (min)	15.32 ± 2.88	15.29 ± 2.63	0.069	0.945
Total duration	8.01 ± 1.47	7.60 ± 1.42	2.144	0.033

### 3.3 Perineal injury

**Figure 1:**
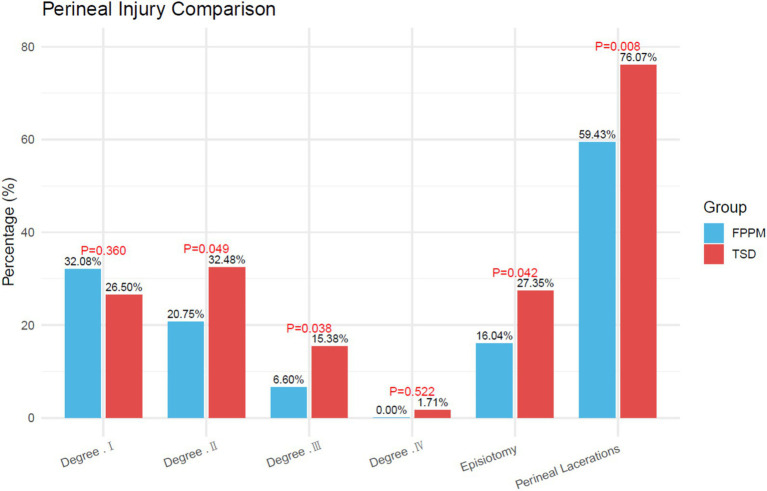
Perineal injury. TSD, Traditional Supine Delivery; FPPM, Free-Position and Perineal Massage.

The incidence of perineal lacerations was significantly lower in the FPPM group compared to the TSD group (59.43% vs. 76.07%; *p* = 0.008; Figure 1). The FPPM group had a lower proportion of degree II (20.75% vs. 32.48%; *p* = 0.049) and degree III lacerations (6.60% vs. 15.38%; *p* = 0.038), as well as a reduced rate of episiotomy (16.04% vs. 27.35%; *p* = 0.042). There were no significant differences between groups in the rates of degree I or degree IV lacerations (*p* = 0.360 and *p* = 0.522, respectively).

### 3.4 Postpartum hemorrhage within 24 h

**Figure 2:**
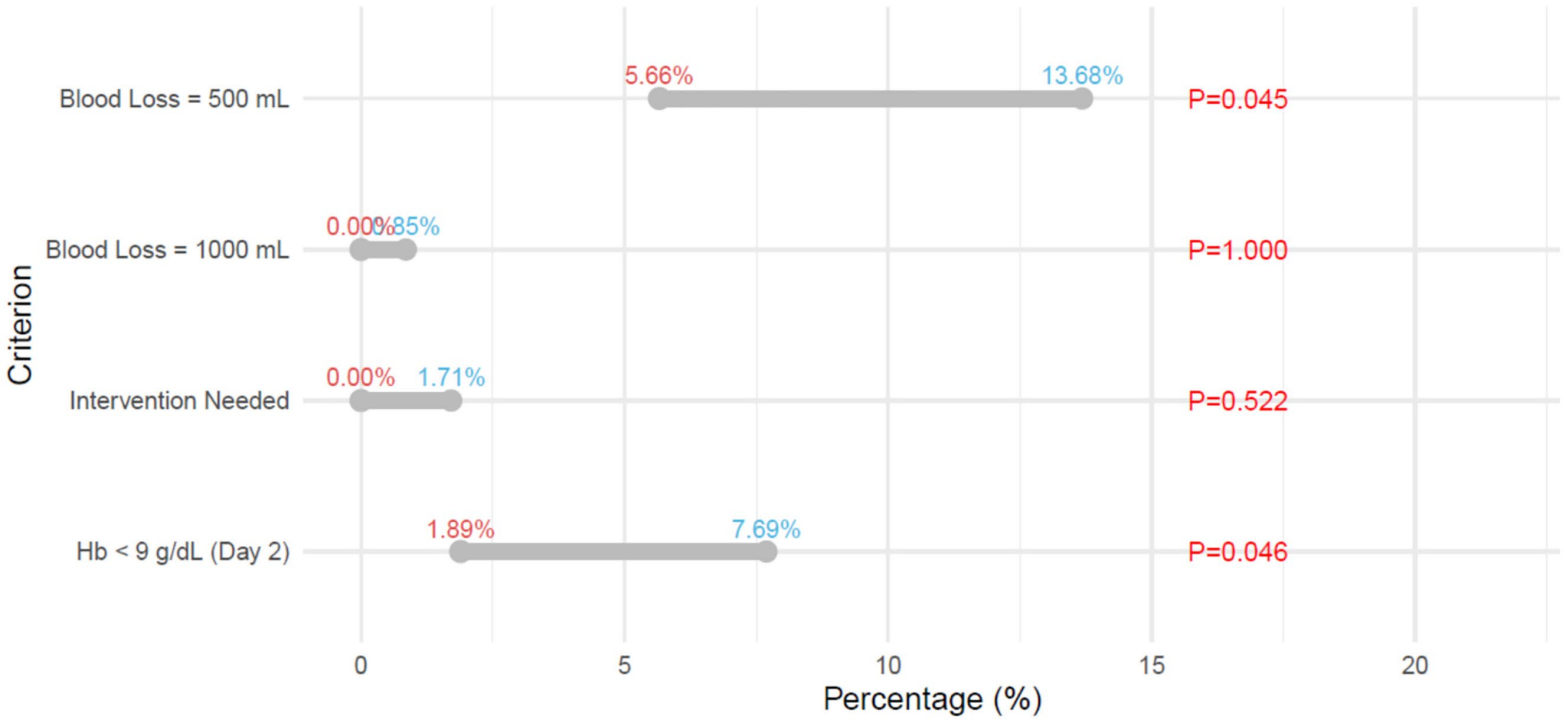
Postpartum hemorrhage within 24 h. Hb, hemoglobin.

Within 24 h postpartum, the incidence of blood loss ≥500 mL was significantly lower in the FPPM group compared with the TSD group (5.66% vs. 13.68%; *p* = 0.045; Figure 2). Similarly, fewer patients in the FPPM group experienced a hemoglobin level on day 2 below 9 g/dL (1.89% vs. 7.69%; *p* = 0.046). There were no significant differences between the groups in the incidence of blood loss ≥1,000 mL or the need for clinical intervention (both *p* > 0.05).

### 3.5 Anesthesia requirements

**Figure 3:**
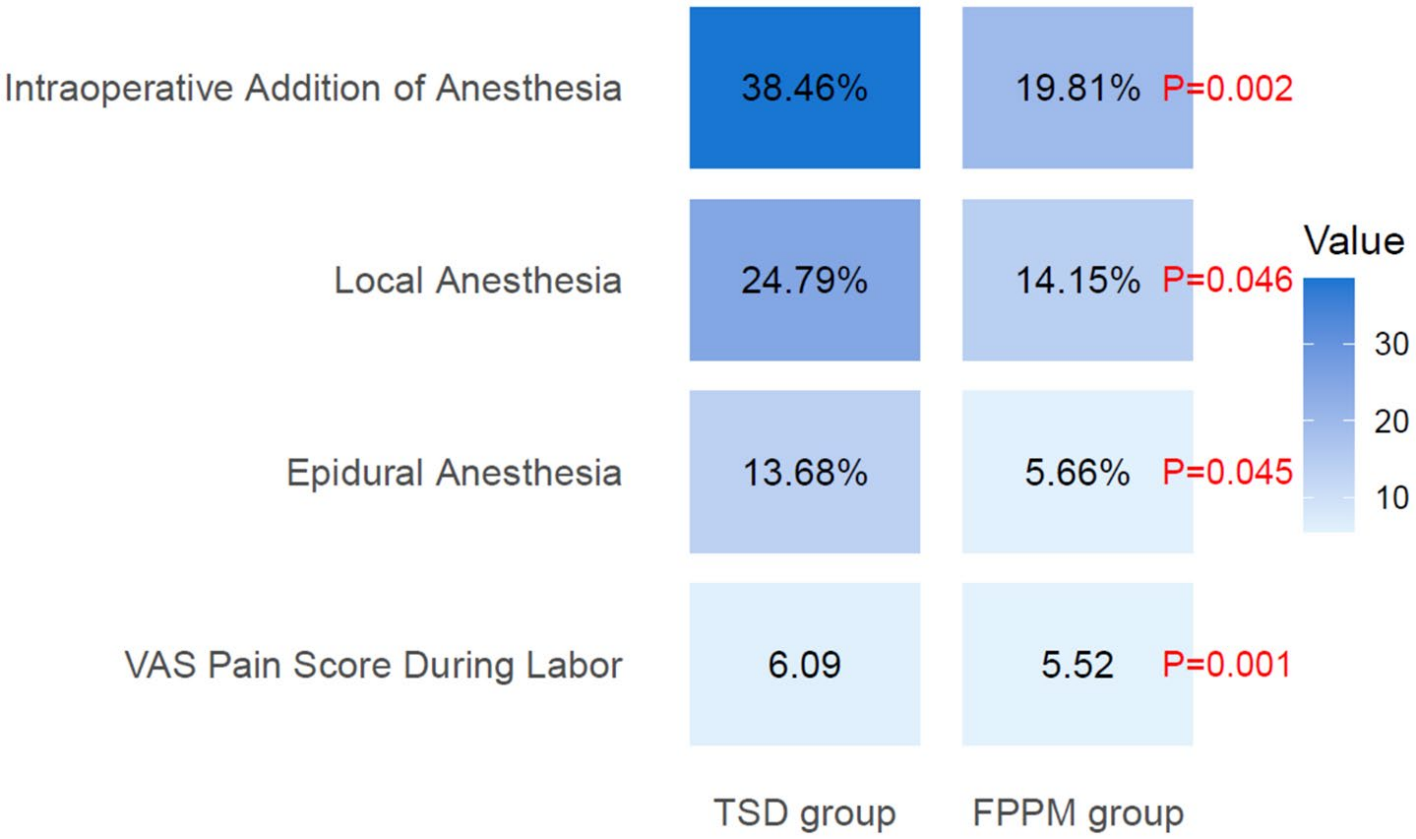
Anesthesia requirements. VAS, Visual Analog Scale.

Anesthesia requirements during labor were generally lower in the FPPM group compared with the TSD group (Figure 3). The FPPM group had a rate of intrapartum addition of anesthesia that was associated with a reduction (19.81% vs. 38.46%; *p* = 0.002), decreased use of local anesthesia that was correlated with a lower frequency (14.15% vs. 24.79%; *p* = 0.046), and less frequent epidural anesthesia administration that was linked to a reduced occurrence (5.66% vs. 13.68%; *p* = 0.045). Additionally, the FPPM group reported VAS pain scores during labor that were associated with lower levels (5.52 ± 1.25 vs. 6.09 ± 1.33; *p* = 0.001).

### 3.6 Postpartum complications

**Figure 4:**
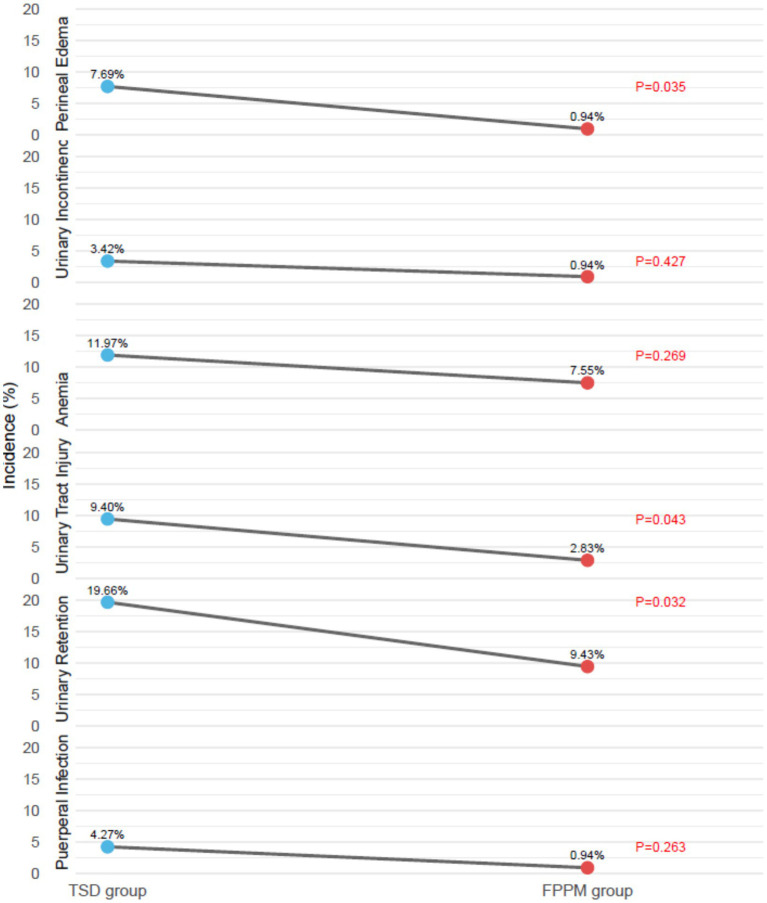
Postpartum complications.

The incidence of postpartum complications was generally lower in the FPPM group compared with the TSD group (Figure 4). Specifically, the FPPM group had rates of urinary retention that were associated with a reduction (9.43% vs. 19.66%; *p* = 0.032), urinary tract injury that were correlated with a decrease (2.83% vs. 9.40%; *p* = 0.043), and perineal edema that were linked to a lower occurrence (0.94% vs. 7.69%; *p* = 0.035). There were no statistically significant differences between the groups in the incidence of puerperal infection, anemia, or urinary incontinence (all *p* > 0.05).

### 3.7 Neonatal condition

Neonatal outcomes were generally favorable in both groups. The FPPM group demonstrated a significantly higher Apgar score at 5 min compared with the TSD group (9.80 ± 0.35 vs. 9.69 ± 0.42; *p* = 0.036; Table 3). There were no significant differences between groups in Apgar score at 1-min, venous cord pH, or neonatal asphyxia rate (all *p* > 0.05).

**Table 3:** Neonatal condition.

Criterion	TSD group (*n* = 117)	FPPM group (*n* = 106)	*t*/*χ*^2^	*p*
Apgar score at 1 minute	9.08 ± 1.17	9.15 ± 0.96	0.490	0.625
Apgar score at 5 minutes	9.69 ± 0.42	9.80 ± 0.35	2.109	0.036
Venous cord pH	7.31 ± 0.05	7.32 ± 0.04	1.714	0.088
Neonatal asphyxia rate	7 (5.98%)	2 (1.89%)	1.468	0.226

### 3.8 Patient psychological status

Regarding psychological outcomes, patients in the FPPM group had significantly lower PCL-5 scores, which were associated with reduced posttraumatic stress symptoms, compared with the TSD group (28.44 ± 2.31 vs. 29.52 ± 2.86; *p* = 0.002). The FPPM group also showed a lower rate of postpartum PTSD tendency, which was correlated with a reduced likelihood of developing PTSD (2.83% vs. 9.40%; *p* = 0.043; Table 4). There were no significant differences between groups in EPDS scores or the rate of postpartum depression tendency (both *p* > 0.05).

**Table 4:** Patient psychological status.

Criterion	TSD group (*n* = 117)	FPPM group (*n* = 106)	*t*/*χ*^2^	*p*
EPDS score	8.32 ± 1.51	8.14 ± 1.37	0.918	0.360
Postpartum depression tendency	17 (14.53%)	10 (9.43%)	1.357	0.244
PCL-5 score	29.52 ± 2.86	28.44 ± 2.31	3.112	0.002
PTSD tendency	11 (9.40%)	3 (2.83%)	4.082	0.043

EPDS, Edinburgh Postnatal Depression Scale; PCL-5, Posttraumatic Stress Disorder Checklist for DSM-5.

### 3.9 Satisfaction

**Figure 5:**
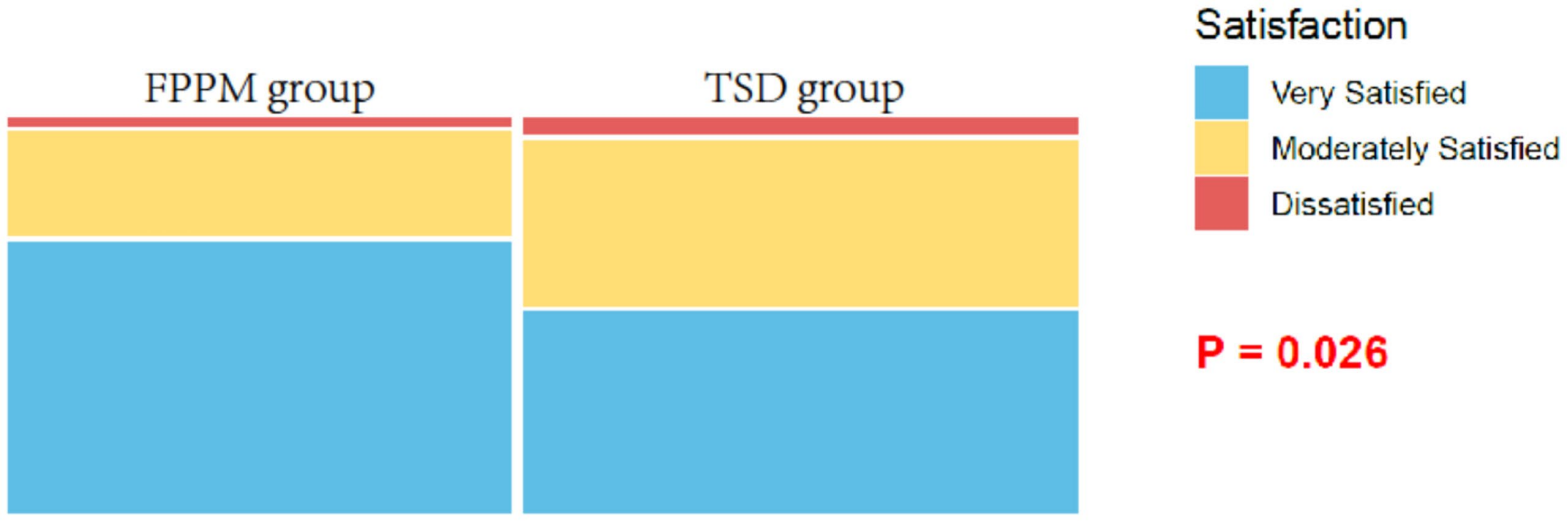
Satisfaction.

Patient satisfaction was significantly higher in the FPPM group compared with the TSD group, with 69.81% of patients reporting being very satisfied versus 52.14% in the TSD group (*p* = 0.026; Figure 5). There were no significant differences in the proportions of patients who were moderately satisfied or dissatisfied between the groups.

### 3.10 Multivariate logistic regression analysis results

A multivariate logistic regression analysis of relevant factors showed that FPPM demonstrated a significant protective effect compared to TSD (*p* = 0.002), indicating that the use of FPPM significantly reduces the risk of perineal lacerations compared to TSD (Table 5). Each additional minute of the second stage of labor was identified as a risk factor (*p* = 0.002). An increase in fetal weight by one unit was also considered a slight risk factor (*p* = 0.022). Similarly, each additional year of maternal age was identified as a risk factor (*p* = 0.025). Pre-pregnancy BMI overweight did not reach statistical significance (*p* = 0.062) but still suggested a potential risk.

**Table 5:** Multivariate logistic regression analysis.

Criterion	Coefficient	*p*	OR	95% CI
FPPM vs. TSD	−0.966	0.002	0.381	0.205–0.707
Second stage duration (min)	0.062	0.002	1.064	1.022–1.107
Fetal weight	0.001	0.022	1.001	1.000–1.001
Maternal age	0.153	0.025	1.166	1.019–1.333
Pre-pregnancy BMI overweight	1.084	0.062	2.957	0.948–9.221

TSD, Traditional Supine Delivery; FPPM, Free-Position and Perineal Massage.

## 4 Discussion

The findings of this study provide new insights into the optimal care strategies for high-risk pregnant women, highlighting the potential advantages of combining free-position delivery with perineal massage. These results should not only be interpreted in terms of direct clinical outcomes but also within the broader physiological, psychological, and organizational mechanisms that support obstetric care. In-depth examination of these underlying factors can further provide a rationale for incorporating such interventions into routine clinical practice while identifying new directions for future research.

The observed reduction in labor duration for women in the FPPM group likely derives from several interrelated influences of maternal mobility and perineal preparation on the mechanics and neurohormonal milieu of labor. Allowing women to adopt positions guided by comfort and instinct (such as upright, lateral, or squatting postures) distinctly contrasts with the conventional supine position, which may impede physiological processes (18). Upright positions exploit gravity, enhance fetal descent, and facilitate uterine contractility through improved uteroplacental perfusion (19). This may be associated with shorter total labor time and a lower incidence of dystocia, which is a significant contributor to maternal and neonatal morbidity in high-risk populations (19). In addition, active mobility may positively influence the axis of the pelvic inlet and outlet, aiding in alignment of the fetus with the birth canal, thereby shortening the active first and second stages (20). Perineal massage, particularly in the late first and second stages, is associated with greater tissue elasticity and increased blood flow, which are related to more compliant perineal tissues during stretching forces exerted in parturition (20).

Mechanistically, perineal massage has been shown to promote collagen remodeling and local tissue heating, increasing extensibility and minimizing microtrauma during crowning and expulsion of the fetus (21). By reducing resistance to fetal passage, this approach is associated with a lower rate and severity of perineal lacerations and a reduced need for mediolateral episiotomy, as observed in the FPPM group (22). Furthermore, the integrated approach may activate gate-control mechanisms of pain modulation, where tactile stimulation serves to attenuate nociceptive signaling, a factor corroborating the reduced VAS pain scores and diminished anesthesia requirements found with FPPM (23). Lower pharmacologic anesthesia exposure aligns with current obstetric safety goals, reducing risks associated with both local and systemic agents, such as maternal hypotension, urinary retention, and delayed ambulation (23).

A notable benefit of the FPPM approach is its association with decreased PPH risk. Reduced rates of blood loss in the FPPM group can be attributed, in part, to a more physiological process of labor and parturition, characterized by improved myometrial efficiency. The avoidance of prolonged labor and traumatic perineal interventions likely lessens myometrial fatigue and the subsequent risk of uterine atony, the leading cause of PPH. Additionally, freedom of movement may provoke endogenous oxytocin release, bolstering uterine contractility in the immediate postpartum period (24, 25). Improved postpartum hemoglobin levels indicate how such non-pharmacologic interventions can ultimately translate into better maternal recovery and lower the need for invasive interventions or transfusions (25).

Another key consideration is the reduction in perineal injury, especially second- and third-degree lacerations, in the FPPM group. The significance of this finding extends beyond immediate postpartum discomfort; it serves to limit long-term pelvic floor dysfunction, urinary incontinence, and sexual health issues, all of which have been documented at higher rates after more severe perineal trauma (26). By proactively maintaining perineal integrity, free-position delivery and perineal massage can contribute to a more positive postpartum experience, both physically and psychologically (27).

The lower incidence of genitourinary complications and perineal edema in the FPPM group is also noteworthy. These outcomes may stem from decreased tissue trauma, but also from enhanced pelvic floor muscle function associated with upright laboring and more physiologic birth mechanics (28). Urinary retention and tract injury are sequelae that can significantly impact postpartum recovery, so minimally invasive perineal care should be emphasized as a quality outcome for high-risk deliveries (28).

Neonatal well-being, as measured by higher five-minute Apgar scores in the FPPM group, may reflect the less stressful birth environment facilitated by maternal mobility and reduced anesthesia use. Enhanced uteroplacental perfusion, more efficient fetal descent, and decreased exposure to anesthetic agents all support robust neonatal adaptation at birth (29). Notably, no differences in more serious outcomes (such as neonatal asphyxia and cord pH) suggest that the approach is safe for infants as well, further supporting its broader adoption.

The psychological benefits observed in the FPPM group, characterized by lower posttraumatic stress scores, reinforce the holistic potential of these interventions. The autonomy in position selection allows mothers to actively manage pain and discomfort, regaining a sense of control over the delivery process, which is a core psychological resource buffering traumatic stress. Perineal massage, as a supportive touch, reduces pain perception through the gate control mechanism and conveys care from healthcare professionals, enhancing the mother’s sense of safety (30). The combination of autonomous decision-making and supportive interaction maintains the mother’s dignity and self-efficacy, counteracting the passivity and objectification often experienced in traditional medical settings, thereby reducing the risk of postpartum PTSD. These aspects may buffer against acute and long-term psychological harm, especially in populations starting with heightened risk profiles due to medical comorbidities or advanced maternal age.

Maternal satisfaction with obstetric care is a comprehensive metric, integrating clinical outcomes, pain and psychological adaptation, fulfillment of birth preferences, and the quality of provider-patient interactions. The higher satisfaction rates in the FPPM group, in the absence of increased adverse outcomes or complications, imply that such interventions align with patient priorities and contemporary standards for respectful, person-centered maternity care. The elements of shared agency, minimized unnecessary intervention, and individualized comfort address powerful determinants of patient satisfaction that are increasingly recognized as core healthcare quality indicators (31).

From a systems perspective, reducing the need for anesthesia, preventing severe perineal injuries and complications, and lowering rates of PPH are associated with alleviating resource pressures on obstetric services. These outcomes are also linked to curbing both direct and indirect costs associated with extended hospital stays or surgical interventions. These findings suggest that the implementation of FPPM protocols for eligible high-risk patients is related to both clinically effective and cost-efficient improvements in existing obstetric practice, particularly when supported by adequate staff training and institutional buy-in.

Physiological theory and recent evidence support these mechanisms. For example, recent studies show that upright positions during labor can enhance uterine contractility and fetal alignment, reducing dystocia and subsequently lowering medical intervention rates (32, 33). Similarly, systematic reviews indicate that antenatal and intrapartum perineal massage decreases the rate of severe perineal trauma and the need for episiotomy, consistent with these findings (33, 34). In addition, gate-control pain theory and studies of maternal mobility support the reduction in pain perception, while research in postpartum mental health highlights the importance of empowerment and autonomy as protective against postnatal psychological distress.

This study included high-risk pregnant women with various risk factors such as advanced maternal age, pregnancy-induced hypertension, and diabetes, aiming to evaluate the overall effectiveness and applicability of free-position delivery combined with perineal massage in a broad high-risk obstetric population. Despite the different pathophysiological mechanisms of these risk factors, the study observed common benefits: shortened labor, reduced perineal trauma, and decreased bleeding risk. These effects may result from universally beneficial mechanisms like optimized pelvic dynamics, improved tissue perfusion and elasticity, and enhanced maternal autonomy. The distribution of various risk factors was balanced between the groups, providing a reliable basis for inter-group comparisons. Future studies need to further explore the specific impacts of the intervention on different subgroups in larger cohorts.

Nevertheless, it is important to acknowledge possible confounding factors. The retrospective study design introduces potential for reporting bias and limits causal inference. This study was conducted at a single center in China and is influenced by local medical resources, the culture of midwife-led care, and variations in maternal acceptance of natural childbirth. These factors limit the generalizability of the results to other cultural contexts and healthcare systems. Staff expertise in administering perineal massage and flexibility in supporting non-supine positions may also be variable across institutions, potentially limiting generalizability. Further, while the findings are compelling for high-risk pregnant women, their applicability to low-risk populations or settings with different resources remains to be established. Future research should focus on addressing the limitations of this study by using prospective designs to reduce reporting bias and enhance causal inference. It is essential to analyze the impact of unmeasured social, cultural, and provider-related factors on care processes and outcomes. Standardizing training for staff in perineal massage and non-supine positions could improve consistency across institutions.

Furthermore, exploring the applicability of these interventions in low-risk populations and diverse resource settings will ensure a comprehensive understanding of best practices in maternity care. Future studies should include neonatal neuroimaging assessments such as cranial ultrasound or MRI as standard observation indicators. This will help to more comprehensively understand the impact of different delivery methods on the newborn’s nervous system, particularly those low-incidence but potentially serious complications, such as asymptomatic intraventricular hemorrhage.

## 5 Conclusion

In conclusion, the integration of free-position during delivery and perineal massage is associated with multifaceted advantages (mechanical, psychological, and organizational) without significant increases in risk to either the mother or the infant. This approach is related to addressing critical indicators of obstetric care quality and advancing maternity practice toward a more physiological, patient-centered paradigm. Continued research is essential to further understand the complex biological and experiential mechanisms involved, optimize protocols for diverse populations, and rigorously assess the impact on both short- and long-term maternal and neonatal outcomes.

## Data Availability

The raw data supporting the conclusions of this article will be made available by the authors, without undue reservation.
